# Hematopoietic Properties of Granulocyte Colony-Stimulating Factor/Immunoglobulin (G-CSF/IgG-Fc) Fusion Proteins in Normal and Neutropenic Rodents

**DOI:** 10.1371/journal.pone.0091990

**Published:** 2014-03-17

**Authors:** George N. Cox, Elizabeth A. Chlipala, Darin J. Smith, Sharon J. Carlson, Stacie J. Bell, Daniel H. Doherty

**Affiliations:** 1 Bolder BioTechnology, Inc., Boulder, Colorado, United States of America; 2 BolderPATH, Inc., University of Colorado, Boulder, Colorado, United States of America; Georg Speyer Haus, Germany

## Abstract

Previously we showed that granulocyte colony-stimulating factor (G-CSF) *in vitro* bioactivity is preserved when the protein is joined via a flexible 7 amino acid linker to an immunoglobulin-1 (IgG1)-Fc domain and that the G-CSF/IgG1-Fc fusion protein possessed a longer circulating half-life and improved hematopoietic properties compared to G-CSF in normal rats. We have extended this analysis by comparing the relative hematopoietic potencies of G-CSF/IgG1-Fc to G-CSF in normal mice and to G-CSF and polyethylene glycol (PEG) - modified G-CSF in neutropenic rats. Mice were treated for 5 days using different doses and dosing regimens of G-CSF/IgG1-Fc or G-CSF and circulating neutrophil levels in the animals measured on Day 6. G-CSF/IgG1-Fc stimulated greater increases in blood neutrophils than comparable doses of G-CSF when administered using daily, every other day or every third day dosing regimens. In rats made neutropenic with cyclophosphamide, G-CSF/IgG1-Fc accelerated recovery of blood neutrophils to normal levels (from Day 9 to Day 5) when administered as 5 daily injections or as a single injection on Day 1. By contrast, G-CSF accelerated neutrophil recovery when administered as 5 daily injections, but not when administered as a single injection. G-CSF/IgG1-Fc was as effective as PEG-G-CSF at accelerating neutrophil recovery following a single injection in neutropenic rats. G-CSF/IgG1-Fc and G-CSF/IgG4-Fc fusion proteins in which the 7 amino acid linker was deleted also were effective at accelerating neutrophil recovery following a single injection in neutropenic rats. These studies confirm the enhanced *in vivo* hematopoietic properties of G-CSF/IgG-Fc fusion proteins.

## Introduction

Granulocyte colony-stimulating factor (G-CSF) is a 19 kDa protein that stimulates the proliferation, differentiation and functional activation of cells of the granulocyte lineage. Recombinant methionyl-G-CSF is widely used to ameliorate neutropenia following myelosuppressive chemotherapy and bone marrow transplantation, and to mobilize peripheral blood progenitor cells for transplantation and blood banking [1], [2]. Recombinant G-CSF is cleared rapidly from the body and typically is administered to patients by daily subcutaneous injection. Cancer patients with neutropenia often receive 10–14 injections of G-CSF per chemotherapy round. Development of longer acting and more potent forms of G-CSF is of considerable interest to patients and healthcare providers.

A number of technologies have been used to improve the pharmacokinetic properties of G-CSF, including modifying the protein with polyethylene glycol (PEG) and fusion of G-CSF to other proteins with long half-lives [3]–[11]. The only longer acting form of G-CSF currently approved for use in humans is a PEG modified form of the protein (Neulasta, Amgen, Inc., reviewed in [12]). PEG modification increases the effective size of G-CSF and slows its rate of absorption from subcutaneous sites and clearance by the kidney and liver. PEG-G-CSF has a 6- to 8-fold longer half-life than G-CSF *in vivo*, which allows the protein to be effective when administered once per chemotherapy cycle (typically once every 3 weeks) to cancer patients [12].

In a previous report we described the construction and preliminary *in vivo* analysis of recombinant fusion proteins comprising G-CSF joined to the Fc (Hinge-CH2-CH3) domains of human IgG1 and IgG4 [10]. The fusion proteins were constructed so that the carboxy-terminus of G-CSF was joined via a flexible 7 amino acid peptide linker to the amino-terminus of the IgG-Fc domain. The fusion proteins were secreted as dimeric proteins from transiently transfected mammalian cells. On a molar basis, the G-CSF/IgG-Fc fusion proteins had biological activities comparable to that of G-CSF in a G-CSF-dependent *in vitro* cell proliferation assay. The fusion proteins possessed ∼8-fold longer half-lives than G-CSF and stimulated greater and longer lasting increases in circulating neutrophils than G-CSF following a single injection in normal rats. Here we build upon these earlier studies by comparing relative *in vivo* potencies of G-CSF and G-CSF/IgG1-Fc in normal mice and G-CSF, PEG-G-CSF and G-CSF/IgG1-Fc in neutropenic rats. The effects of IgG isotype and the requirement for the flexible peptide linker on *in vitro* and *in vivo* bioactivities of the G-CSF fusion proteins also are explored.

## Materials and Methods

### Ethics statement

All animal experiments were carried out in strict accordance with the recommendations in the Guide for the Care and Use of Laboratory Animals of the National Institutes of Health. Protocols were approved by BolderPATH's Institutional Animal Care and Use Committee. To alleviate pain, rats and mice were anesthetized by inhalation of isoflurane prior to taking blood samples. Animals were euthanized by CO_2_ inhalation.

### Construction of plasmids encoding G-CSF/IgG-Fc fusion proteins and expression of the proteins in mammalian cells

Plasmids encoding G-CSF/IgG1-Fc and G-CSF/IgG4-Fc fusion proteins containing a 7 amino acid peptide linker (SerGlyGlySerGlyGlySer) joining G-CSF to the IgG-Fc domains have been described [10]. New fusion protein gene constructs in which the peptide linker is deleted were created using PCR mutagenesis methods [13]. Transient transfection of monkey COS-1 cells (American Type Culture Collection, Manassas, VA) with plasmids encoding the fusion proteins and purification of the secreted fusion proteins from conditioned media by protein A affinity chromatography were performed essentially as previously described [10]. Concentrations of the purified proteins were measured using a Bradford dye binding assay kit (Bio-Rad Laboratories, Richmond, CA) using bovine serum albumin as the protein standard. *In vitro* bioactivities of the fusion proteins were measured in a 3-day cell proliferation assay using the mouse NFS-60 cell line [10]. *In vitro* bioactivity experiments were performed in triplicate wells for each protein dilution and were repeated at least 3 times for each protein. Recombinant human methionyl-G-CSF (R & D Systems, Inc.) was used as the G-CSF standard in the bioassays.

Protein samples for purity analyses were prepared in sodium dodecyl sulfate polyacrylamide gel electrophoresis (SDS-PAGE) sample buffer with (reducing) or without (non-reducing) the addition of 1-5% (v/v) 2-mercaptoethanol, electrophoresed on precast Tris-glycine polyacrylamide gels (Invitrogen Corporation, Carlsbad, CA) and stained with Coomassie Blue (Bio-Rad Laboratories).

### Normal mouse efficacy experiments

Female ICR mice, weighing 15–20 g at study initiation, were obtained from Harlan-Sprague Dawley (Indianapolis, IN). Animals were acclimated for at least 7 days before study initiation. One group of 5 mice was sacrificed on Day 0 to provide an untreated control group complete blood cell count (CBC). The remaining mice were randomized to different test groups, consisting of 5 mice each. Mice received subcutaneous injections of vehicle solution (phosphate buffered saline containing 100 μg/mL mouse serum albumin (Sigma-Aldrich, Inc., St. Louis, MO), methionyl-G-CSF (Neupogen, Amgen, Inc.) or G-CSF/IgG1-Fc according to different dosing schedules. The protein concentration of Neupogen and the fusion proteins were measured using the Bradford dye binding assay utilizing bovine serum albumin as a standard. Animals were treated using an every day dosing regimen (injections on Days 1–5), an every other day dosing schedule (injections on Days 1, 3 and 5) or an every third day dosing schedule (injections on Days 1 and 4). On Day 6 the animals were sacrificed and a terminal blood sample was obtained using EDTA anticoagulant tubes. A portion of the blood samples was sent to Antech Diagnostics, Inc. (Irvine, CA) for CBC analyses. Statistical analyses between groups were done by comparing group means using the Student's t-test with significance set at p≤0.05. The spleen and sternum were removed, weighed, and fixed in 10% neutral buffered formalin for histopathologic evaluation. Relative spleen weight was calculated by multiplying the spleen weight by 100 and dividing by the body weight. The sternums were decalcified in 5% formic acid and processed along with the spleens for histopathologic evaluation of sections stained with hematoxylin and eosin. Bone marrow and spleen sections were examined without prior knowledge of the treatment. Spleen sections were examined qualitatively. Bone marrow sections were examined under low and high power for qualitative differences in numbers of myeloid and erythroid precursor cells in order to obtain an estimate of the myeloid:erythroid cell ratio (M:E ratio). In some experiments, M:E ratios in bone marrow sections were determined quantitatively by performing an approximate 200 cell differential count. Cells were counted at 400× magnification along a linear guide in the ocular field of view. Approximately 3–5 random fields were counted from different vertebrate levels to ensure random sampling until 200 cells were obtained. Statistical analyses between groups were done by comparing group means using the Student's t-test with significance set at p≤0.05.

### Neutropenic rat efficacy experiments

Sprague-Dawley rats, weighing ∼200 g, were obtained from Harlan-Sprague Dawley. Rats were allowed to acclimate for at least 7 days before study initiation. Rats were randomized to different test groups based upon their Day 0 neutrophil counts. There were 5 rats per test group. All but one of the test groups received an intraperitoneal dose of 100 mg/kg of cyclophosphamide (CPA) on Day 0 to induce neutropenia. One group of control rats did not receive CPA. During the next 1–5 days the different test groups received subcutaneous injections of vehicle solution (phosphate buffered saline), 100 μg/kg methionyl-G-CSF (Neupogen, Amgen, Inc.), 100 μg/kg of various G-CSF/IgG-Fc fusion proteins, or 100 μg/kg PEG-methionyl-G-CSF (Neulasta, Amgen, Inc.), using different dosing schedules depending upon the study design. The protein concentration of Neulasta, Neupogen and the fusion proteins were measured using the Bradford dye binding assay utilizing bovine serum albumin as a standard. Protein samples were prepared in vehicle solution. Blood samples (0.4 mL) were collected from the rats using EDTA anticoagulant tubes on Day 0, Days 1–10 and Day 12 and sent to Antech Diagnostics to perform CBC analyses. Statistical analyses between groups were done by comparing group means using the Student's t-test with significance set at p≤0.05

## Results

### Construction, expression and *in vitro* bioactivities of G-CSF/IgG direct fusion proteins

**Figure 1:**
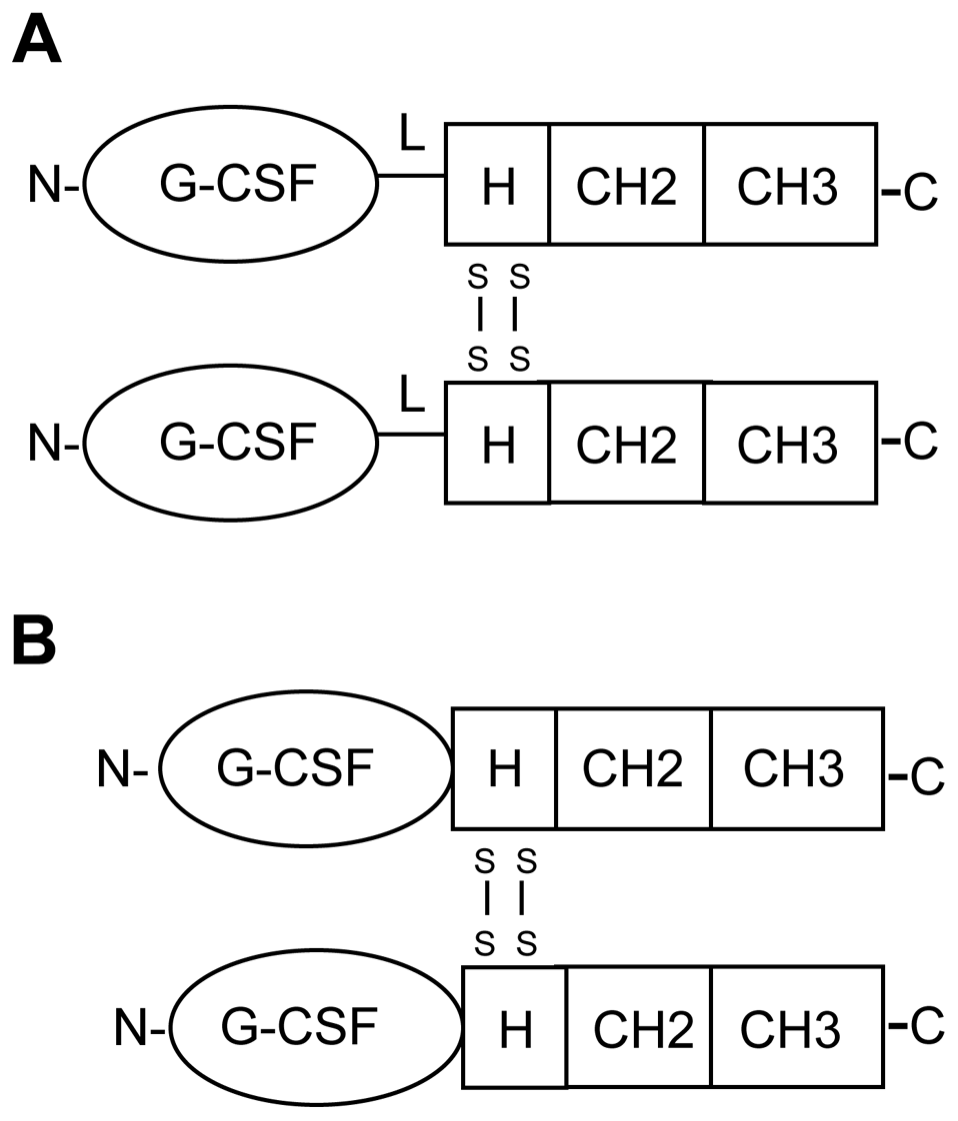
Schematic diagram of (A) G-CSF/IgG-FcL (linkered) and (B) G-CSF/IgG-FcD (direct) fusion proteins. In the linkered constructs, the carboxy-terminus of G-CSF is joined via a seven amino acid linker (L) to the amino terminus of IgG1-Fc and IgG4-Fc domains. In the D (direct) constructs, the carboxy-terminus of G-CSF is joined directly to the amino terminus of IgG1-Fc and IgG4-Fc domains. The hinge (H), CH2 and CH3 regions of the IgG-Fc fragments are indicated. The fusion proteins are dimeric due to disulfide bonds (S-S) that form between cysteine residues located in the IgG Hinge region.

**Figure 2:**
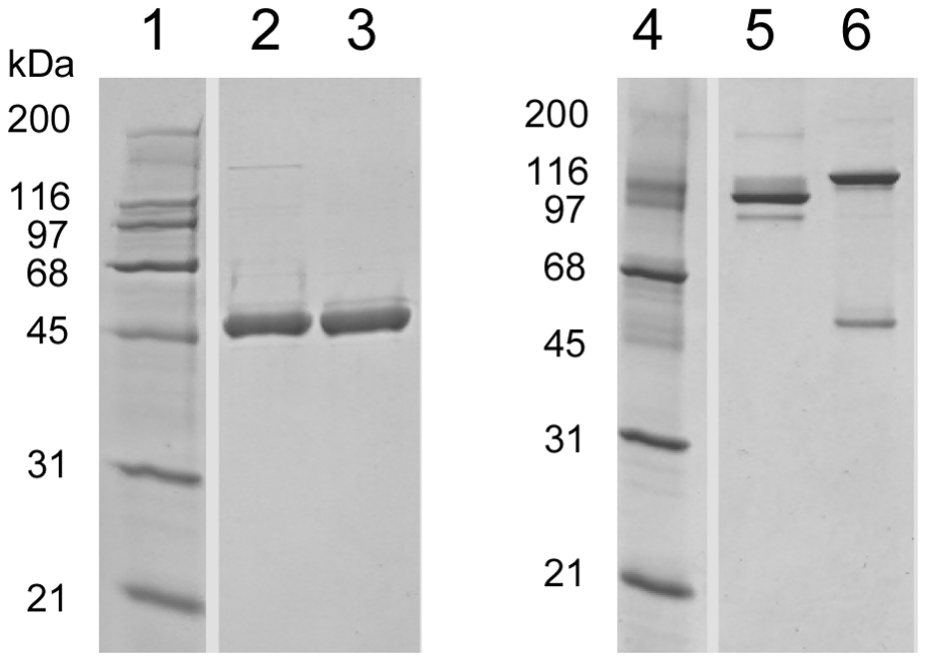
SDS-PAGE analysis of purified G-CSF/IgG-Fc direct fusion proteins. Lanes 1-3 are reducing SDS-PAGE and lanes 4-6 are non-reducing SDS-PAGE. Lanes 1 and 4 are molecular weight markers; lanes 2 and 5 are G-CSF/IgG1-FcD; and lanes 3 and 6 are G-CSF/IgG4-FcD.

The construction of genes encoding G-CSF/IgG-Fc fusion proteins in which the carboxy-terminus of human G-CSF is joined via a 7 amino acid peptide linker (SerGlyGlySerGlyGlySer) to the amino-terminus of the Fc domain of human IgG1 or IgG4 was described previously [10]. On a G-CSF molar basis these fusion proteins had similar *in vitro* bioactivities as G-CSF, as measured by proliferation of the G-CSF–dependent mouse NFS-60 cell line. Because the peptide linkers are non-natural amino acids and could potentially be immunogenic in humans new genes encoding fusion proteins in which the carboy-terminal amino acid of G-CSF (Proline-174) is joined directly to the amino-terminal amino acid (Glutamic acid-1) of the IgG1-Fc and IgG4-Fc domains were constructed [14], [15]. The constructs containing linkers are referred to as “L” or linker fusion constructs and the fusions containing no linkers are referred to as “D” or direct fusion constructs (Fig. 1). The G-CSF/IgG-FcD fusion proteins were expressed by transient transfection of monkey COS cells and purified from conditioned media by Protein A affinity chromatography. The G-CSF/IgG-FcD fusion proteins were recovered principally as disulfide-linked homodimers (Fig. 2), similar to the G-CSF/IgG-FcL fusion proteins described previously [10]. Dimerization of the proteins presumably occurs through disulfide bonds formed between cysteine residues located in the Hinge regions of the IgG-Fc domains (Fig. 1). Like the purified G-CSF/IgG-FcL fusion proteins, the purified G-CSF/IgG-FcD fusion proteins also had similar apparent molecular weights of about 50,000 as estimated by reducing SDS-PAGE and 110,000 (IgG1 fusion protein) and 120,000 (IgG4 fusion protein) as estimated by non-reducing SDS-PAGE (Fig. 2). The larger apparent molecular weight of the G-CSF/IgG4-FcD protein compared to the G-CSF/IgG1-FcD protein by non-reducing SDS-PAGE suggests that the G-CSF/IgG4-FcD protein has a larger hydrodynamic radius than the G-CSF/IgG1-Fc protein, which may be due to the conformational and segmental flexibility differences between IgG1-Fc and IgG4-Fc domains [16]. The G-CSF/IgG4-Fc fusion protein also contained a minor 50,000 molecular weight protein species when analyzed by non-reducing SDS-PAGE (Fig. 2B). The 50,000 molecular weight protein appears to be monomeric fusion protein, which may derive from non-disulfide bonded dimers. IgG4 antibodies typically contain small amounts of non-disulfide bonded dimers, which result from the two hinge region cysteine residues forming intramolecular rather than intermolecular disulfide bonds [17]. The G-CSF/IgG-FcD and G-CSF/IgG-FcL fusion proteins possessed similar *in vitro* bioactivities, as measured by their ability to stimulate proliferation of NFS-60 cells. The EC_50_s for the G-CSF/IgG1-FcD and G-CSF/IgG4-FcD fusion proteins in this bioassay were 45±12 and 47±12 pg/mL, respectively, similar to the values reported previously for G-CSF/IgG1-FcL (38.3±4.0 pg/mL) and G-CSF/IgG4-FcL (56.7±5.9 pg/mL) [10]. The control met-G-CSF protein had an EC_50_ of 22±1 pg/mL in this bioassay, similar to the EC_50_ value of 17.7±0.6 ng/mL reported previously by us in the NFS60 cell assay [10]. On a G-CSF molar basis the G-CSF/IgG-Fc D and L fusion proteins and met-G-CSF had similar *in vitro* bioactivities, since G-CSF contributes about 40% of the mass of the fusion proteins. These data indicated that G-CSF/IgG-Fc *in vitro* bioactivity is not appreciably affected by the presence or absence of the peptide linker joining G-CSF to the IgG-Fc domains.

### Relative hematopoietic potencies of G-CSF/IgG1-FcL and G-CSF in normal mice

To gain a better understanding of the relative *in vivo* potencies of G-CSF and G-CSF/IgG-Fc fusion proteins, we compared the hematopoietic properties of recombinant G-CSF (Neupogen, Amgen, Inc.) and a representative G-CSF/IgG-Fc fusion protein, G-CSF/IgG1-FcL, in normal mice, employing methods similar to those used by others to measure relative potencies of various G-CSF analogs [18]. We compared potencies of the proteins using every day, every other day or every third day subcutaneous dosing regimens. Mice were injected subcutaneously with different doses of G-CSF/IgG1-FcL and a fixed dose of G-CSF over the course of 5 days and the animals sacrificed on Day 6 for CBC analyses. Control animals were injected with vehicle solution (phosphate buffered saline containing 100 μg/mL mouse serum albumin).

Mice treated every day (Days 1–5) with 0.8, 4, 20 or 100 μg/kg of G-CSF/IgG1-FcL showed dose-dependent increases in circulating neutrophils, white blood cells and lymphocytes (Table 1). Mice treated with 100 μg/kg of G-CSF/IgG1-FcL had nearly 10-fold higher number of circulating neutrophils than mice treated with the same dose of G-CSF. It should be noted that on a molar basis, mice receiving 100 μg/kg of G-CSF received approximately 2.5 times as much G-CSF as the mice receiving 100 μg/kg G-CSF/IgG1-FcL since G-CSF accounts for only about 40% of the mass of the fusion protein. Mice treated with 20 μg/kg of G-CSF/IgG1-FcL had greater numbers of circulating neutrophils than mice treated with 100 μg/kg of G-CSF, suggesting that G-CSF/IgG1-FcL is at least 5-fold more potent than G-CSF on a mass basis. Platelet levels in the mice showed a dose-dependent decrease in response to G-CSF or G-CSF/IgG1-FcL treatment (Table 1). None of the proteins caused a significant change in red blood cell counts in the mice (Table 1). Histological analysis of bone marrow samples taken from untreated mice and vehicle treated mice on Day 6 showed myeloid and erythroid precursor cells in approximate equal numbers, as expected for normal mice (M:E ratio of ∼1, Table 1). In contrast animals treated with G-CSF/IgG1-FcL or G-CSF showed elevated bone marrow M:E ratios on Day 6 (Table 1), indicating continued stimulation of granulopoiesis at this time. The M:E ratio was highest in animals treated with 100 μg/kg G-CSF/IgG1-FcL and mirrored the blood neutrophil data. Animals treated with G-CSF or the 20 and 100 μg/kg doses of G-CSF/IgG1-FcL had significantly enlarged spleens (Table 1) relative to control and vehicle treated animals. Histological analysis of spleen sections from these animals revealed elevated extramedullary granulopoiesis and increased numbers of mature neutrophils in the red pulp. Absolute spleen weight and relative spleen weight (spleen weight adjusted for body weight) correlated with the degree of granulopoiesis and numbers of mature neutrophils.

**Table 1:** Effect of every day (days 1-5) dosing of G-CSF and G-CSF/IgG1-FcL on day 6 blood cell counts in mice.

Treatment	Dose	PMN	WBC	RBC	Platelets	Lymphs	M:E	Spleen
	(μg/kg)	(10^3^/μL)	(10^3^/μL)	(10^6^/uL)	(10^3^/uL)	(10^3^/μL)	Ratio	(mg)
Untreated	-	0.8±0.1	5.7±0.9	8.5±0.3	980±46	4.7±0.8	1.1±0.1	121±19
Vehicle	-	0.6±0.1	5.7±1.0	8.3±0.2	900±29	4.7±0.9	1.0±0.1	113±17
G-CSF	100	3.9±0.6^a^	11.5±0.7^a^	8.8±0.5	568±50^a^	7.3±0.5^a^	12.6±3.5	194±33
G-CSF/IgG1-Fc	100	31.9± 2.4^a, b^	45.8±2.7^a, b^	7.9±0.4	537±26^a^	12.8±1.3^a, b^	22.0±5.1	258±23^a^
G-CSF/IgG1-Fc	20	5.8±0.9^a^	14.2±2.0^a^	8.8±0.3	663±12^a^	7.8±1.2^a^	9.1±1.1	200±29^a^
G-CSF/IgG1-Fc	4	1.1±0.2^b^	8.2±0.7^b^	8.6±0.3	1,007±115^b^	6.9±0.6^a^	1.9±0.2	122±7
G-CSF/IgG1-Fc	0.8	0.9±0.1	8.5±1.1^b^	9.0±0.2	950±40^b^	7.3±1.1	1.1±0.1	113±9

Mice received a subcutaneous injection every day for 5 days with vehicle solution, or the indicated doses of G-CSF or G-CSF/IgG1-FcL. On day 6 blood samples were obtained from the mice and the number of neutrophils (PMN), white blood cells (WBC), red blood cells (RBC), platelets, and lymphocytes (Lymphs) was determined. On day 6 the animals were sacrificed, their spleens weighed and the ratio of myeloid to erythroid cells (M: E ratio) in sections of the bone marrow determined. Blood and tissue samples were obtained from untreated mice on Day 1. Data are means ± SE for 5 mice per group. ^a^p≤0.05 versus untreated controls; ^b^p≤0.05 versus G-CSF.

Mice receiving every other day (Days 1, 3 and 5) injections of G-CSF/IgG1-FcL also showed dose-dependent increases in circulating neutrophils, white blood cells and lymphocytes on Day 6 (Table 2). Mice receiving every other day injections of 100 μg/kg of G-CSF/IgG1-FcL had greater circulating levels of neutrophils than mice treated every other day or every day with 100 μg/kg of G-CSF (Table 2). Analysis of bone marrow sections taken from the animals on Day 6 showed elevated M:E ratios in bone marrow samples of animals treated with the two highest doses of G-CSF/IgG1-FcL or G-CSF (Table 2). The bone marrow of these animals was dominated by mature granulocytes. Animals treated with the 100 μg/kg EOD dose of G-CSF/IgG1-FcL had significantly enlarged spleens relative to control and vehicle treated animals (Table 2). Histological analysis of spleen sections from these animals revealed elevated extramedullary granulopoiesis and increased numbers of mature neutrophils in the red pulp. Untreated mice and vehicle treated mice had minimal to mild splenic extramedullary hematopoiesis characterized primarily by the presence of erythoid precursors and megakaryocytes.

**Table 2:** Effect of every other day (days 1, 3 and 5) dosing of G-CSF and G-CSF/IgG1-FcL on day 6 blood cell counts in mice.

Treatment	Dose	PMN	WBC	RBC	Platelets	Lymphs	M:E	Spleen
	(μg/kg)	(10^3^/μL)	(10^3^/μL)	(10^6^/uL)	(10^3^/uL)	(10^3^/μL)	Ratio	(mg)
Untreated	-	0.7±0.1	5.7±0.9	7.2±0.7	640±93	4.7±0.7	1.0	132±14
G-CSF (ED)	100	4.1±0.8^a^	12.3±1.2^a^	7.9±0.6	679±58	7.9±0.6^a^	9.0	127±11
Vehicle (EOD)	-	0.9±0.1	6.7±0.3	8.4±0.1	1,026±57^a^	5.4±0.3	1.0	104±8
G-CSF (EOD)	100	1.6±0.3^a, b^	7.3±1.2^b^	7.4±0.9	825±109	5.5±0.9	2.3	117±2
G-CSF/IgG1-Fc (EOD)	100	11.2±1.7^a, b^	19.5±2.6^a, b^	8.7±0.2^a^	587±28	7.6±0.8^a^	49.0	207±28^a^
G-CSF/IgG1-Fc (EOD)	20	2.6±0.3^a, b^	10.1±1.3^b, c^	7.0±0.8	679±51	7.0±1.0	4.0	135±7
G-CSF/IgG1-Fc (EOD)	4	1.0±0.2^b^	7.6±1.4^b^	8.3±0.2^b^	905±20	6.2±1.1	1.0	141±14
G-CSF/IgG1-Fc (EOD)	0.8	1.0±0.1^b^	7.9±0.9^b^	7.1±1.1	875±113	6.6±0.8	1.0	130±6

Mice received subcutaneous injections every day (ED; on Days 1–5) or every other day (EOD; on Days 1, 3 and 5) with vehicle solution, or the indicated doses of G-CSF or G-CSF/IgG1-FcL. On day 6 blood samples were obtained from the mice and the number of neutrophils (PMN), white blood cells (WBC), red blood cells (RBC), platelets, and lymphocytes (Lymphs) was determined. On day 6 the animals were sacrificed, their spleens weighed and the ratio of myeloid to erythroid cells (M: E ratio) in sections of the bone marrow determined. Blood and tissue samples were obtained from untreated mice on Day 1. Data are means ± SE for 5 mice per group. ^a^p≤0.05 versus untreated controls; ^b^p≤0.05 versus G-CSF.

Every third day (Days 1 and 4) administration of 100 μg/kg of G-CSF/IgG1-FcL or G-CSF to mice did not significantly increase blood neutrophil levels above those seen in animals receiving vehicle solution, although bone marrow M:E ratios were mildly elevated in the animals on Day 6 (Table 3). However, every third day administration of a larger dose of G-CSF/IgG1-FcL (300 μg/kg) stimulated a significant increase in blood neutrophil counts on day 6, whereas the same dose of G-CSF did not. M:E ratios were higher in bone marrow samples of animals treated with 100 μg/kg and 300 μg/kg of G-CSF/IgG1-FcL than in animals receiving the same doses of G-CSF (Table 3). Absolute spleen weights and relative spleen weights were significantly elevated in animals receiving 300 μg/kg of G-CSF/IgG1-FcL. Histological analysis of spleen samples revealed that the extent of extramedullary granulopoiesis was greatest in animals treated ETD with G-CSF/IgG1-FcL 300 μg/kg, followed by animals treated ED with G-CSF 100 μg/kg, followed by animals treated ETD with 100 μg/kg G-CSF/IgG-Fc or ETD with 300 μg/kg of G-CSF. The extent of splenic granulopoiesis was similar in the untreated, vehicle and the 100 μg/kg ETD G-CSF treatment groups.

**Table 3:** Effect of every third day (dosing on days 1 and 4) dosing of G-CSF and G-CSF/IgG1-FcL on day 6 blood cell counts in mice.

Treatment	Dose	PMN	WBC	RBC	Platelets	Lymphs	M:E	Spleen
	(μg/kg)	(10^3^/μL)	(10^3^/μL)	(10^6^/uL)	(10^3^/uL)	(10^3^/μL)	Ratio	(mg)
Untreated	-	0.6±0.1	3.8±0.9	8.0±0.7	611±140	3.1±0.8	1.2	120±10
G-CSF (ED)	100	3.0±0.4^a^	9.2±0.6^a^	8.5±0.1	495±082	5.9±0.4^a^	15.4	190±10^a^
Vehicle (ETD)	-	0.9±0.2^b^	5.6±0.9^b^	8.2±0.2	652±207	4.4±0.8^b^	1.2	130±10
G-CSF (ETD)	300	0.9±0.2^b^	7.4±0.5^b^	8.7±0.2	565±144	6.2±0.6^b^	3.5	170±10
G-CSF (ETD)	100	1.2±0.2^b^	8.7±1.0^a^	8.7±0.1	982±076	7.1±0.9^a^	3.2	150±10
G-CSF/IgG1-Fc (ETD)	300	8.0±2.3^a^	17.6±3.3^a, b^	8.1±0.2	492±138	8.8±1.0^a, b^	25.9	240±10^a^
G-CSF/IgG1-Fc (ETD)	100	1.3±0.2^b^	7.6±0.8	8.7 ± 0.1	831±107^b^	5.9±0.7	8.3	180±10^a^

Mice received subcutaneous injections every day (ED; on Days 1–5) or every third day (ETD; on Days 1 and 4) with vehicle solution, or the indicated doses of G-CSF or G-CSF/IgG1-FcL. On day 6 blood samples were obtained from the mice and the number of neutrophils (PMN), white blood cells (WBC), red blood cells (RBC), platelets, and lymphocytes (Lymphs) was determined. On day 6 the animals were sacrificed, their spleens weighed and the ratio of myeloid to erythroid cells (M: E ratio) in sections of the bone marrow determined. Blood and tissue samples were obtained from untreated mice on Day 1. Data are means ± SE for 5 mice per group. ^a^p≤0.05 versus untreated controls; ^b^p≤0.05 versus G-CSF.

### Relative hematopoietic potencies of G-CSF/IgG-Fc fusion proteins, G-CSF and PEG-G-CSF in neutropenic rats

**Figure 3:**
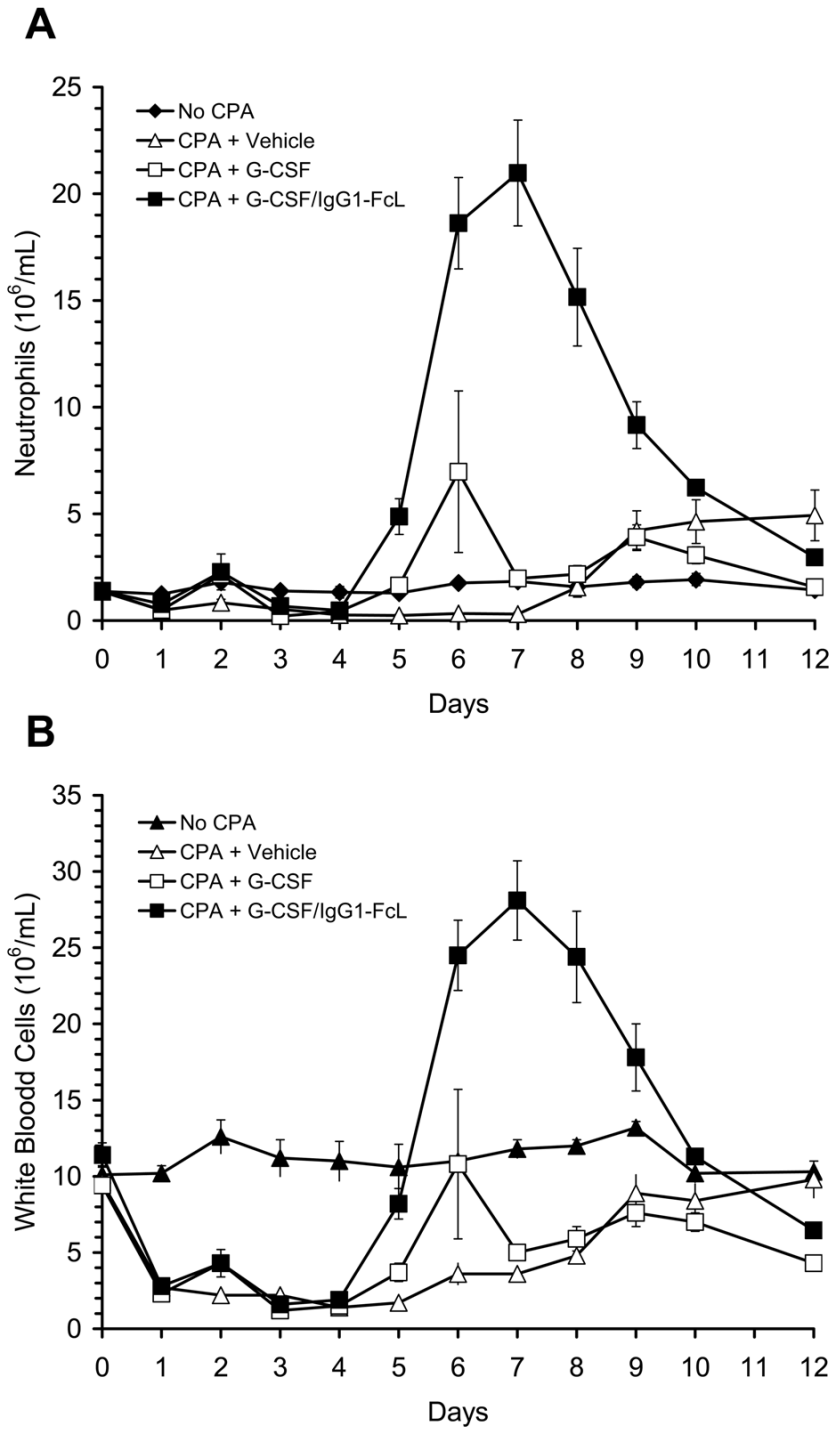
Changes in neutrophil and white blood cell counts in neutropenic rats treated daily with G-CSF/IgG1-FcL. Rats were made neutropenic by injection of cyclophosphamide (CPA) on Day 0. Beginning on Day 1 and continuing through Day 5 different groups of rats received daily injections of G-CSF (100 μg/kg), G-CSF/IgG1-FcL (100 μg/kg) or vehicle solution. The No CPA control group did not receive CPA but did receive injections of vehicle solution on Days 1 through 5. Blood samples were obtained from the rats on the days indicated and neutrophil (**Panel A**) and white blood cell (**Panel B**) levels were measured. Data are means ± SE for 5 rats/group.

The primary clinical use for G-CSF is the acceleration of neutrophil recovery following myelosuppressive chemotherapy in cancer patients [1], [2]. We compared the ability of G-CSF and various G-CSF/IgG-Fc fusion proteins to accelerate neutrophil recovery in chemotherapy (CPA)-treated rats [19] as a model for chemotherapy-induced neutropenia in cancer patients. Rats, rather than mice, were used for these studies because of their larger size, which allowed us to monitor changes in neutrophil and white blood cell counts in the same animals over time and thus greatly reduced the number of animals required for the studies. In an initial experiment we compared the ability of G-CSF and a representative G-CSF/IgG-Fc fusion protein, G-CSF/IgG1-FcL, to accelerate recovery from CPA-induced neutropenia in rats when the proteins were administered daily for 5 days beginning on Day 1 following CPA treatment. This dosing regimen mimics the daily dosing regimen typically used for G-CSF in cancer patients. Rats were given an intraperitoneal injection of CPA (100 mg/kg) on day 0 to induce neutropenia. G-CSF/IgG1-FcL or G-CSF was injected subcutaneously into the rats at a dose of 100 μg/kg on Days 1–5. Control animals received injection of vehicle solution. One control group of animals did not receive CPA on Day 0, but did receive injections of vehicle solution on Days 1-5. Circulating neutrophil and white blood cell counts in the animals were measured from Day 0 to Day 12 and are shown in Fig. 3. CPA-treatment caused a rapid drop in white blood cell and neutrophil levels during the first 2 days following treatment. Red blood cell and platelet levels also decreased in the CPA-treated animals (see below). A transient increase in circulating neutrophil and white blood cell levels occurred on Day 2 in all animals receiving G-CSF or G-CSF/IgG1-FcL. Neutrophil and white blood cell counts in the animals decreased to a nadir on Day 4. In neutropenic animals receiving vehicle solution circulating neutrophil levels did not return to Day 0 levels until Day 8 or Day 9 post-CPA treatment. The rebound in neutrophil counts was followed by a period of neutrophilia until study termination on Day 12. In animals treated with G-CSF or G-CSF/IgG1-FcL, neutrophil counts returned to normal levels by Day 5 post-CPA treatment, significantly faster than in animals treated with vehicle solution. Temporal changes in white blood cell counts for the different test groups were qualitatively similar to the neutrophil data. On Days 5-9, neutrophil and white blood cell counts in G-CSF/IgG1-FcL-treated animals were significantly higher than in animals treated with the same dose of G-CSF. None of the treatments significantly accelerated recovery of red blood cell or platelet levels in the animals (data not shown). Platelet levels in the rats reached a nadir around Day 4 or 5 post-CPA treatment and recovered to baseline levels by Day 7 or 8, irrespective of treatment. Red blood cell levels reached a nadir around Day 7 or 8 post-CPA-treatment and had not fully recovered to baseline levels by study termination on Day 12.

**Figure 4:**
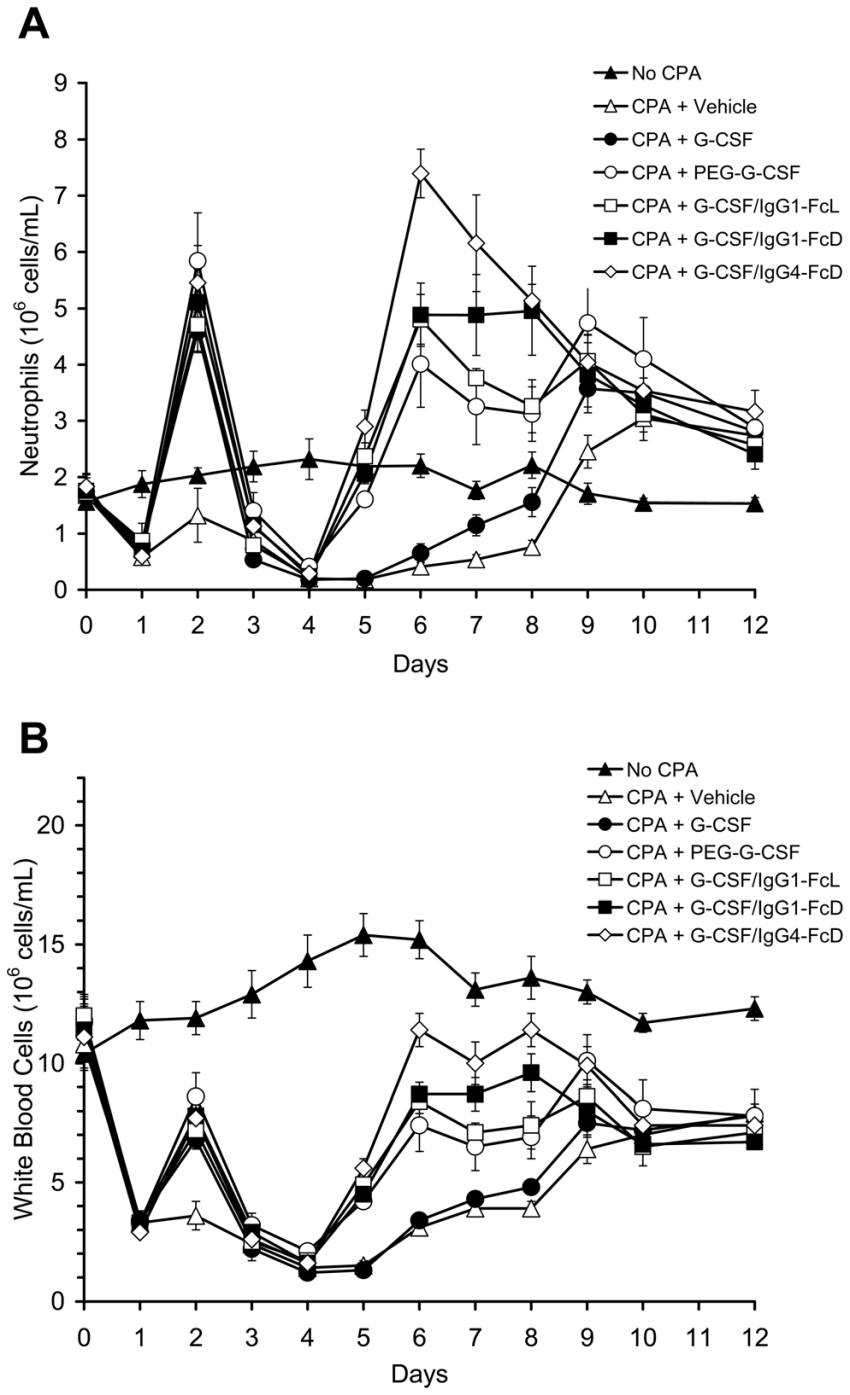
Changes in neutrophil and white blood cell counts in neutropenic rats treated once with G-CSF/IgG-Fc proteins. Rats were made neutropenic by injection of CPA on Day 0. On Day 1 different groups of rats received injections of G-CSF (100 μg/kg), PEG-G-CSF (100 μg/kg), G-CSF/IgG1-FcL (100 μg/kg), G-CSF/IgG1-FcD (100 μg/kg), G-CSF/IgG4-FcD (100 μg/kg), or vehicle solution. The No CPA control group did not receive CPA but did receive an injection of vehicle solution on Day 1. Blood samples were obtained from the rats on the days indicated and neutrophil (**Panel A**) and white blood cell (**Panel B**) levels measured. Data are means ± SE for 5 rats/group.

In a follow-up experiment we asked whether a single injection of G-CSF or G-CSF/IgG1-Fc given on Day 1 following chemotherapy would be sufficient to accelerate neutrophil recovery in CPA-treated rats. This dosing regimen mimics the dosing regimen currently used for the long-acting G-CSF analog, PEG-G-CSF (Neulasta, Amgen, Inc.) in cancer patients [8], [12]. PEG-G-CSF was included as a comparator in this study, since it is the current gold standard long-acting G-CSF therapy. The G-CSF/IgG-FcD and G-CSF/IgG4-FcD fusion proteins also were included in this experiment to determine if there were significant differences in *in vivo* bioactivities of the different G-CSF/IgG-Fc fusion proteins in this clinically relevant model. The design of this study was identical to that described above except that the rats received only a single injection of the test proteins (100 μg protein/kg) on Day 1 following CPA treatment. Control rats received a single injection of vehicle solution. As shown in Fig. 4, animals receiving any of the G-CSF/IgG-Fc fusion proteins, G-CSF or PEG-G-CSF displayed a transient increase in neutrophils and white blood cells on Day 2 post-injection. Neutrophil and white blood cell counts reached a nadir on Day 4 in the CPA-treated rats. A single injection of G-CSF did not accelerate recovery of neutrophils or white blood cells compared to vehicle in CPA-treated animals (Fig. 4). Circulating neutrophil levels in both groups returned to baseline values by about Day 9 post-CPA-treatment. White blood cell counts in both groups showed a similar, gradual increase from the nadir beginning on Day 6, but never recovered to baseline values. In contrast to the results obtained with G-CSF, a single injection of PEG-G-CSF caused neutrophil levels to return to baseline levels between Days 5 and 6 post-CPA treatment, confirming the greater in vivo potency of PEG-G-CSF. A single injection of any of G-CSF/IgG-Fc fusion proteins also caused neutrophil counts to return to normal levels by about Day 5, similar to PEG-G-CSF. Animals treated with G-CSF/IgG4-FcD had the highest neutrophil counts on Days 5-8. Neutrophil levels were significantly (p<0.05) higher on Days 5-8 in animals treated with G-CSF/IgG4-FcD compared to animals treated with PEG-G-CSF. White blood cell levels in the PEG-G-CSF treatment group and all of the G-CSF/IgG-Fc treatment groups began to increase by Day 5 and the increase was more rapid than in vehicle- or G-CSF-treated animals. White blood cell counts in the PEG-G-CSF treatment group and all of the G-CSF/IgG-Fc treatment groups were significantly higher than white blood cell levels in vehicle-treated animals on days 5-8. Only animals treated with the G-CSF/IgG4-FcD fusion protein recovered white blood cell levels to Day 0 values. By Day 10 white blood cell levels were similar in all test groups treated with CPA, and this level was below the Day 0 level, largely reflecting a deficit in lymphocytes (Fig. 4B and data not shown). None of the treatments caused significant acceleration of red blood cell or platelet levels in the animals (data not shown).

## Discussion

These studies confirm and extend our previous finding that the fusion of G-CSF to human IgG-Fc domains does not appreciably affect G-CSF *in vitro* bioactivity, but does significantly increase G-CSF potency *in vivo* [10]. Whether G-CSF is joined directly or via an intervening peptide linker to IgG-Fc domains appears to have little effect on *in vitro* or *in vivo* bioactivities of the fusion proteins. G-CSF has a 4-helix bundle structure shared by many cytokines and growth factors [20], [21]. Regions of G-CSF that interact with the G-CSF receptor have been localized to helices 1 and 4 and the amino-terminal end of the loop region joining helices 1 and 2 [22]-[25]. Helix 4 of G-CSF extends to within two amino acids of the C-terminus of the protein [20]. The close proximity of this receptor binding helix to the C-terminus of G-CSF is what prompted us initially to construct G-CSF/IgG-Fc fusions containing the 7 amino acid flexible linker [10]. We hypothesized that the linker would reduce the probability that the IgG-Fc domain would adversely affect G-CSF binding to its receptor and the fusion protein's bioactivity. Data presented here indicate that G-CSF/IgG-Fc fusion proteins with and without the intervening peptide linker have similar *in vitro* bioactivities, indicating that the linker is superfluous and not necessary to preserve G-CSF bioactivity. These data support the notion that the extreme carboxy-terminus of G-CSF is dispensable for G-CSF bioactivity. This conclusion is also supported by the finding that large PEGs can be attached to the C-terminus of G-CSF without significantly altering G-CSF *in vitro* bioactivity [11]. These data also confirm our previous finding [10] that dimerization does not appreciably diminish G-CSF *in vitro* or *in vivo* bioactivity.

G-CSF/IgG1-FcL was used as a representative G-CSF/IgG-Fc fusion protein to compare relative *in vivo* potencies of G-CSF/IgG-Fc fusions with G-CSF in normal mice and neutropenic rats. Titration experiments in mice using different doses of G-CSF/IgG1-FcL and a fixed dose of G-CSF suggested that only about 20% as much G-CSF/IgG1-FcL by weight was required to produce equivalent increases in circulating neutrophils and white blood cells as G-CSF using an every-day dosing regimen. G-CSF/IgG1-FcL was more effective than G-CSF at stimulating granulopoiesis in mice when less frequent dosing regimens were employed, presumably due to its longer half-life. Whereas G-CSF was able to stimulate increases in Day 6 neutrophil levels in mice when administered every day (on Days 1-5) it was not effective when administered every other day (on Days 1, 3 and 5) or every third day (on Days 1 and 4). In contrast, G-CSF/IgG-FcL stimulated Day 6 neutrophil levels when administered every day, every other day or every third day.

G-CSF/IgG1-FcL also proved significantly more potent than G-CSF at accelerating recovery from neutropenia in CPA-treated rats, which is a model for chemotherapy-induced neutropenia in cancer patients, the largest clinical indication for G-CSF. G-CSF typically is administered to cancer patients by daily sc injection beginning 24 hr following chemotherapy and continuing for 10–14 days until neutrophil counts have returned to normal levels [8], [12]. When similar doses of G-CSF and G-CSF/IgG1-FcL were administered daily for 5 days to CPA-treated rats, animals receiving either protein had their neutrophil levels return to normal levels by Day 5 post-CPA, approximately 3-4 days faster than animals treated with vehicle solution. Although time to neutrophil recovery was similar in animals treated with G-CSF and G-CSF/IgG1-FcL, the magnitude of the neutrophil response was significantly greater in rats receiving G-CSF/IgG1-FcL. Rats receiving G-CSF/IgG1-FcL had 10-fold greater circulating neutrophil counts on Day 6 than animals treated with G-CSF, and neutrophil levels remained elevated for a longer period of time. The data suggest that lower doses of G-CSF/IgG1-FcL could be used in this model to produce results comparable to those produced by G-CSF, although such studies have not been performed.

PEG-G-CSF has captured a dominant share of the cancer neutropenia market due to its more convenient once per chemotherapy cycle dosing regimen [12]. We compared relative efficacies of all 3 G-CSF/IgG-Fc fusion proteins to PEG-G-CSF in the single injection rat neutropenia study since this model most closely mimics how PEG-G-CSF is used in the clinic and provides the best test for relative potencies of the different fusion proteins to PEG-G-CSF. We confirmed that a single injection of PEG-G-CSF was sufficient to accelerate neutrophil recovery in the rat CPA-induced neutropenia model, similar to the situation in cancer patients. A single injection of any of the G-CSF/IgG-Fc fusion proteins accelerated neutrophil recovery at least as well as the same dose of PEG-G-CSF in this model. By contrast, a single injection of G-CSF showed little benefit versus vehicle solution, confirming the need for repeated injections of G-CSF for efficacy in this model. The ability to effectively treat chemotherapy patients with a single injection of a drug rather than multiple daily injections offers patients a more convenient treatment option and ensures patient compliance over an extended period. The rodent efficacy data presented here suggest that all 3 G-CSF/IgG-Fc fusion proteins would be effective in cancer patients using a once per chemotherapy cycle dosing regimen, similar to the dosing regimen used for PEG-G-CSF.

The G-CSF/IgG1-FcL protein has a 5- to 8-fold longer half-life than G-CSF, which accounts for its greater *in vivo* potency compared to G-CSF [10]. Although pharmacokinetic studies were not performed with the new G-CSF/IgG-Fc direct fusion proteins, it is likely that they also have extended half-lives compared to G-CSF since they proved as efficacious *in vivo* as G-CSF/IgG1-FcL in the single injection rat neutropenia study. The G-CSF/IgG-Fc direct fusion proteins contain only human protein sequences and thus, at least theoretically, have less potential for being immunogenic in humans than the fusion proteins containing the non-natural linker sequences. For this reason, the direct fusion proteins may be preferred human clinical development candidates. Testing immunogenicity of the different fusion proteins is beyond the scope of this study, as it would require performing human clinical trials with multiple G-CSF/IgG-Fc fusion proteins. Although immunogenicity of the different G-CSF/IgG-Fc fusion proteins could be compared in animals, such studies are of limited value because animals typically develop antibodies to human proteins and the results are not always predictive of immunogenicity of the proteins in humans [26]. The enhanced hematopoietic properties of G-CSF/IgG-Fc fusion proteins makes them useful tools for studying G-CSF function *in vivo* and may lead to the development of a new class of long-acting G-CSF analogs for treating human diseases.
